# The concordance and correlation of Global Burden of Disease stroke incidence rates with stroke incidence rates from population-based stroke registers

**DOI:** 10.1177/17474930261423425

**Published:** 2026-02-03

**Authors:** Daniel Youkee, Charles DA Wolfe

**Affiliations:** 1 Stroke Research Group, Neurology Division, Neuroscience Institute, University of Cape Town, Cape Town, South Africa; 2 School of Life course and Population Health Sciences, King’s College London, London, UK

**Keywords:** Register, incidence, burden, population, global burden of disease, stroke

## Abstract

**Background:**

The Global Burden of Disease (GBD) 2021 study and population-based stroke registers are principal sources of stroke incidence estimates. This study aims to assess the concordance and correlation between GBD stroke incidence rates and stroke incidence rates from population-based stroke registers.

**Methods:**

Crude and age-standardized stroke incidence rates were sourced from high-quality population-based stroke registers and compared to GBD estimates matched by year and location, using GBD subnational data where available. Studies were categorized by country income group status using the World Bank country classifications: high-income countries (HICs); upper middle-income countries (UMICs), and lower middle-income countries (LMICs). Studies were categorized as to whether they were reported as informing the GBD 2021 model, using the online GBD 2021 sources tool. Concordance and correlation were assessed using Lin’s concordance correlation coefficient and Pearson’s correlation coefficient, respectively. Bland–Altman plots were created to display 95% limits of agreement.

**Findings:**

Fifty crude matched incidence rates and 31 matched age-standardized rates were compared. Concordance and correlation for crude stroke incidence were 0.67 and 0.68 overall, 0.66 and 0.68 for HICs, 0.59 and 0.77 for UMICs and 0.03 and 0.97 for LMICs respectively. Overall, 11 (22.0%) GBD estimates, accounting for UIs, matched population-based stroke register crude incidence rates. 95% limits of agreement were -110.2/100,00 to 134.1/100,000 overall. Concordance and correlation for age-standardized incidence rates were 0.56 and 0.59 overall, 0.59 and 0.63 for HICs, 0.12 and 0.17 for UMICs, and 0.25 and 0.42 for LMICs. 95% limits of agreement were from −94.6 to 84.1/100,000. Subgroup analysis including only studies where more specific subnational geographical GBD estimates were available marginally improved crude incidence (n = 18) concordance (0.67 to 0.71) but not age-standardized incidence (n = 13) concordance (0.53 to 0.49). Subgroup analysis limited to population-based stroke registers included as GBD 2021 sources, did not significantly improve correlation or concordance.

**Interpretation:**

Our findings demonstrate limited concordance and correlation in crude and age-standardized stroke incidence rates between population-based stroke registers and the GBD 2021 model, with lower concordance for UMICs and LMICs, compared to HICs. The wide 95% limits of agreement demonstrated should provide caution in the use of GBD stroke incidence estimates to guide policy or assess progress in the primary prevention of stroke.

## Graphical abstract

**Graphical abstract:**
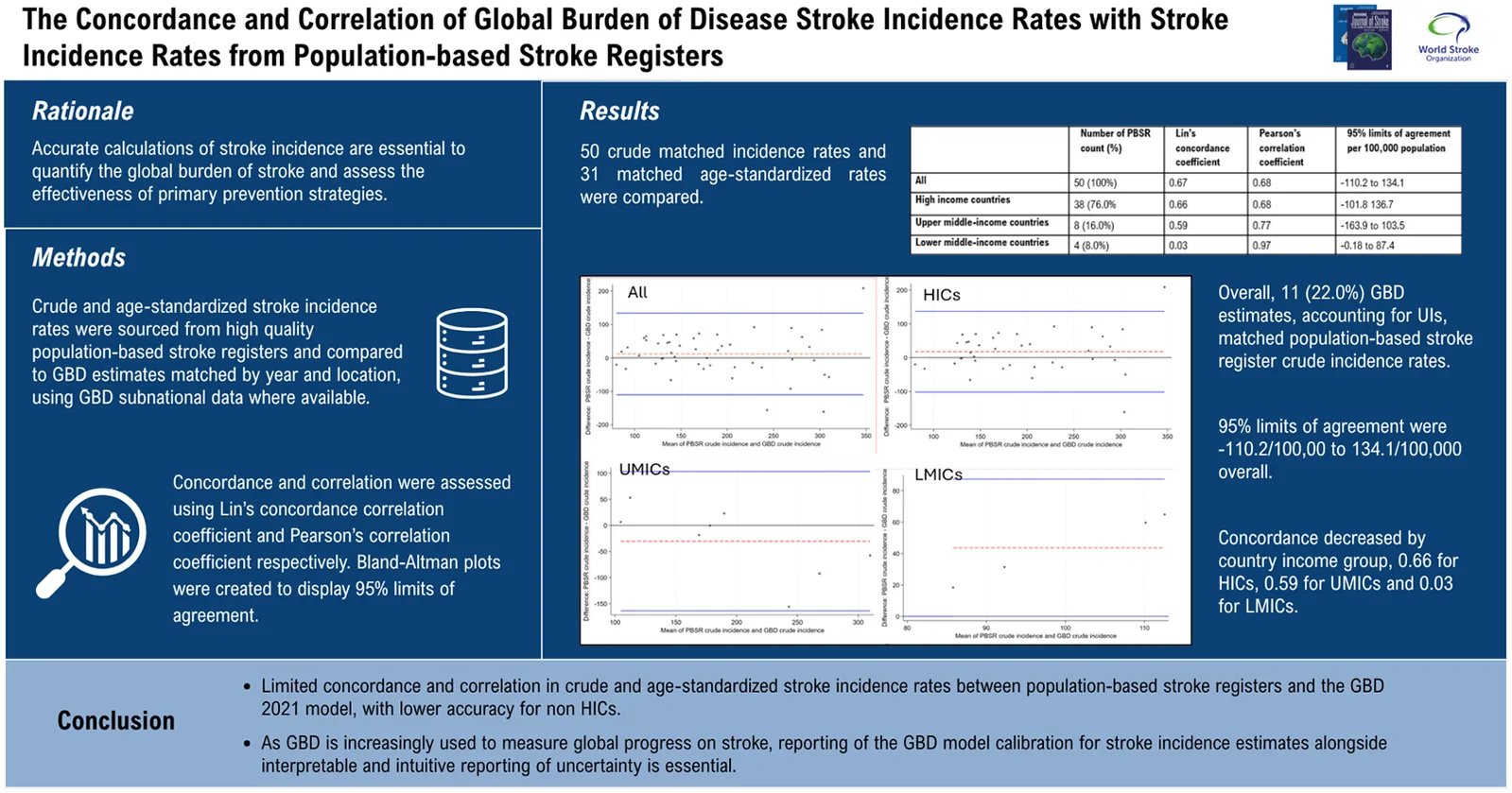

## Introduction

Accurate calculations of stroke incidence are essential to quantify the global burden of stroke and assess the effectiveness of primary prevention strategies. Since the 1980s, population-based stroke registers have been vital sources of stroke incidence data.^1,2^ Population-based stroke registers have measured trends in stroke incidence over time,^3^ by socioeconomic status,^4^ ethnic group,^5^ gender,^6^ and stroke subtypes.^7^ Methodological criteria of population-based stroke registers has been developed over time to improve comparability and standardization of case ascertainment.^8^ Population-based stroke registers measure stroke incidence with high accuracy for specific locations, however these populations may not be representative of the national population.

Stroke incidence measurements from population-based stroke registers are incorporated into the Global Burden of Disease (GBD) Study 2021, this predictive model estimates stroke incidence for 204 countries and territories from 1990 to 2021.^9^ The GBD 2021 stroke incidence estimates were published in October 2024 and has been cited by 467 articles as of September 2025. The GBD model estimates stroke incidence for every location, even when data are highly inconsistent or there are no data for a disease or risk, a best estimate is produced along with a best estimate of uncertainty.^10^ This approach derives from the logic that a best estimate borrowing insight from where data are available is better than no estimate, provided that there is clarity around the level of uncertainty.^10^ GBD reports uncertainty intervals (UIs), to characterize uncertainty surrounding estimates, previously for conditions other than stroke GBD UIs have been reported as counterintuitive to common understandings of the availability and quality of source data.^11,12^

A standard step in predictive model development and validation is assessing model calibration, the agreement between observed and predicted outcome values.^13,14^ The calibration of the GBD 2021 Stroke model is not reported. Therefore, this analysis aims to assess GBD model calibration by analyzing the concordance and correlation between predicted GBD 2021 stroke incidence estimates and observed stroke incidence measurements from population-based stroke registers. A separate analysis explores stroke incidence uncertainty intervals (UIs) reported by the GBD 2021 model, compared to the availability and number of source data utilized by GBD 2021 by region and by the absolute number of stroke incidence reported by GBD.

## Methods

### Data sources

Population-based stroke registers measuring crude and age-standardized stroke incidence were identified from previous systematic reviews of population-based stroke registers and stroke incidence.^1,15^ Population-based stroke registers that met the following criteria were included: stroke defined as the World Health Organization definition; first-ever stroke in a life-time; complete community-based ascertainment (hospital and non-hospital sources); multiple overlapping sources; supplementary methods of case ascertainment, including referrals for imaging and follow-up of patients referred with transient ischemic attack; prospective design using hot pursuit; population base should be large and stable; and time period should include whole years (because of the seasonality of stroke). Studies with lower rates of neuroimaging than the “ideal” criteria of 90% were included and rates of neuroimaging are reported by study. Population-based stroke registers providing data from 1990 onwards, the start date of GBD estimates, until 2021 were included. We excluded purely community-based surveys which did not include active hospital-based case ascertainment and retrospective reviews of hospital discharge summaries or ICD codes. Population-based stroke registers were categorized by country income group using the World Bank country classifications: high-income countries (HICs); upper middle-income countries (UMICs); lower middle-income countries (LMICs); and low-income countries(LICs). Population-based stroke registers were categorized by United Nations geographical region; Africa; Asia; Europe; Latin America and Caribbean; Northern America; and Oceania. Population-based stroke registers were categorized as to whether they were reported as informing the GBD 2021 model, using the online GBD 2021 sources tool.

Crude incidence rates and age-standardized incidence rates, standardized to the WHO World Standard population where available, were extracted from the population-based stroke registers and compared to GBD estimates of incidence matched by year and location. GBD estimates of crude and age-standardized stroke incidence were extracted from the VizHub repository,^16^ on 13 September 2025, along with corresponding 95% uncertainty intervals (UIs), independently by two researchers. GBD estimates were matched to the year and country of location of the population-based stroke register. For multi-year studies, the midpoint year of the population-based stroke register was used. For locations where GBD provides disaggregated data by region the closest geographical location to the population-based stroke register was used.

The main analysis compares crude stroke incidence rates from the two data sources. Concordance and correlation of crude stroke incidence rates from the two data sources were assessed using Lin’s concordance correlation coefficient and Pearson’s correlation coefficient respectively for all studies, by study country income group, by geographical region, by whether the study was included in the GBD 2021 model and by whether subnational GBD data was available. Bland–Altman plots were created to display bias and 95% limits of agreement. A sub analysis of concordance and correlation of age-standardized incidence rates from the same sample of population-based stroke registers, standardized to the WHO World Standard population, to the GBD 2021 model was conducted.

A separate analysis to investigate the association of GBD UIs with the number of data sources informing the estimate and the value of the estimate was conducted. The number of data sources cited by GBD 2021^9^ for incidence of first-ever ischemic stroke were categorized by region^9^ and 21 estimates of regional first-ever ischemic stroke incidence rates and corresponding UIs were extracted from VizHub.^16^ The width of GBD UIs were compared between regions with no data sources informing the GBD 2021 model to regions with one or more data sources, using unpaired t-tests. Width of GBD UIs for crude incidence estimates were compared with the value of the GBD crude incidence estimates, using paired t-tests.

Statistical analysis was conducted in STATA v18.0.

## Results

Fifty population-based stroke registers were included, see supplemental Table 1. For each of the 50 population-based stroke register crude incidence measurements, 50 estimates were extracted from the GBD data repository, matched by year and location, see supplemental Table 1. Thirty-eight (76.0%) of population-based stroke registers were conducted in high-income countries, 8 (16.0%) in upper middle-income countries, and 4 (8.0%) in lower middle-income countries (see Table 1). Population-based stroke registers were conducted in Africa (2), Asia (9), Europe (27), Latin America and Caribbean (5), North America (2), and Oceania (6). Subnational-level GBD estimates were available for 18 (36%) of studies and the comparison was made with national rates for the remaining 32 (64%). Forty-five (90%) of the studies were reported as informing the GBD 2021 model.

**Table 1.** Concordance, correlation, and 95% limits of agreement for crude incidence rates by country income group, region, studies with corresponding subnational GBD estimates only and studies reported as informing GBD estimates only.

	Number of PBSR count (%)	PBSR crude incidence (SD) per 100,000 population	GBD crude incidence (SD) per 100,000 population	Difference in agreement:	PBSR crude incidence falls within GBD UI: count (%)	Lin’s concordance coefficient	Pearson’s correlation coefficient	95% limits of agreement per 100,000 population
All	50 (100%)	193.8 (75.8)	181.9 (79.6)	11.9	11 (22.0%)	0.67	0.68	-110.2 to 134.1
High-income countries	38 (76.0%	203.9 (79.5)	186.4 (71.6)	17.5	9 (23.7%)	0.66	0.68	-101.8 136.7
UMICs	8 (16.0%)	181.8 (53.3)	212.0 (100.0)	-30.2	2 (25.0%)	0.59	0.77	-163.9 to 103.5
LMICS	4 (8.0%)	122.0 (24.3)	78.4 (2.1)	43.6	0 (0.0%)	0.03	0.97	-0.18 to 87.4
Africa	2 (4.0%)	101.5 (9.2)	76.6 (0)	24.9	0 (0.0%)	-	-	-
Asia	9 (18.0%)	182.4 (49.4)	198.2 (112.5)	-15.8	2 (22.2%)	0.50	0.69	-184.8 to 153.2
Europe	27 (54.0%)	223.6 (83.9)	211.1 (66.6)	12.5	5 (18.5%)	0.63	0.66	-113.6 to 138.7
Latin America and Caribbean	5 (10.0%)	135.2 (43.7)	116.2 (14.1)	19.0	1 (20.0%)	0.26	0.55	-55.3 to 93.3
North America	2 (4.0%)	166.5 (37.5)	141.8 (10.5)	24.7	2 (100.0%)	-	-	-
Oceania	6 (12.0%)	160.0 (28.8)	118.8 (12.5)	41.2	1 (20.0%)	0.08	0.34	-12.1 to 94.6
Studies with corresponding Subnational GBD estimates only	18 (36.0%)	183.9 (64.2)	156.7 (58.6)	27.2	6 (33.3%)	0.71	0.78	- 53.1 to 107.4
Studies reported as informing GBD estimates	45 (90.0%)	190.7 (77.5)	175.7 (75.8)	15	11 (24.4%)	0.69	0.71	-100.0 to 130.0

PBSR = population-based stroke register.

**Figure 1.**
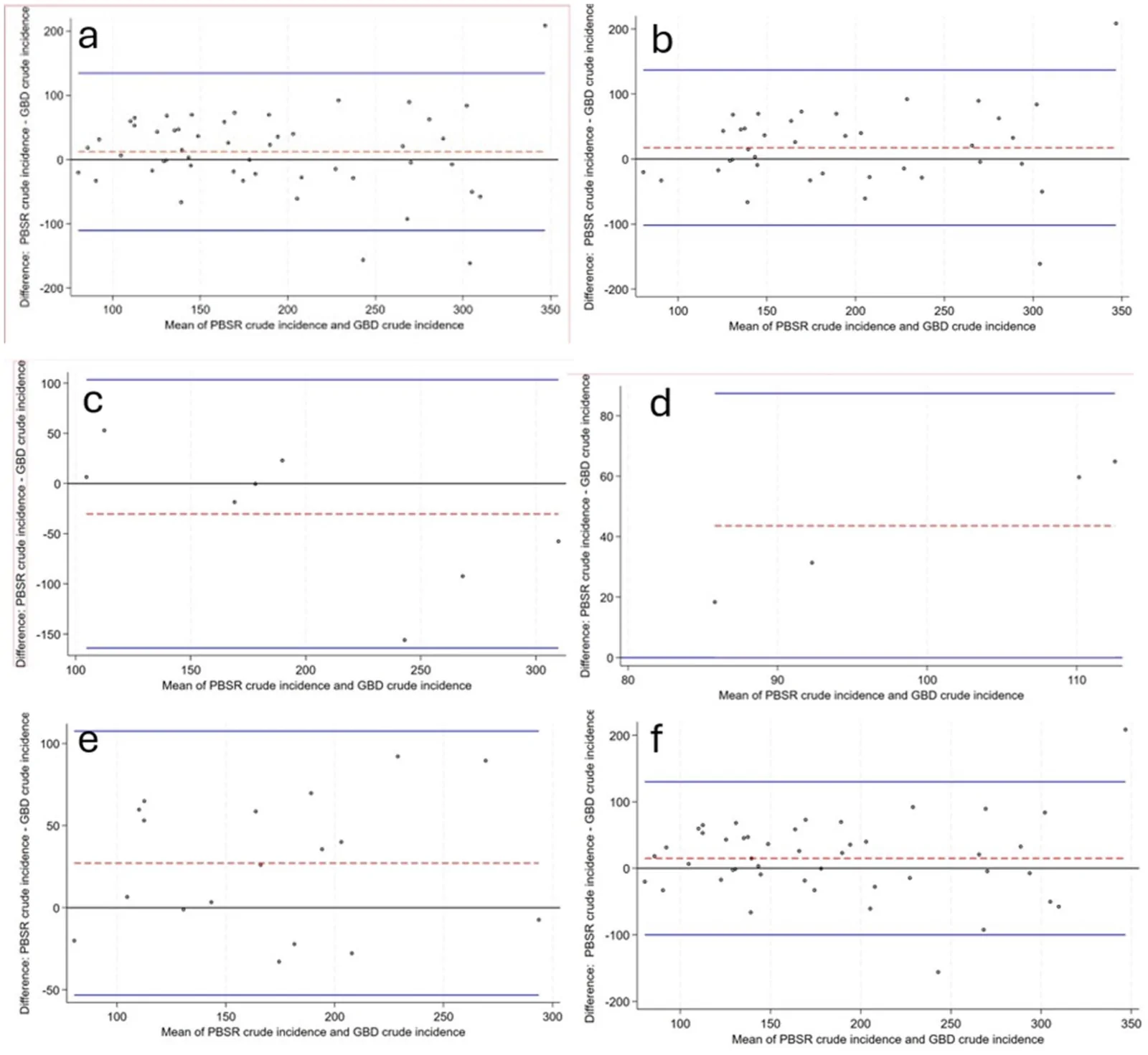
Bland–Altman plots of crude incidence from population-based stroke registers compared to GBD crude incidence estimates per 100,000 population and 95% limits of agreement: (a) all studies (n = 50) (b) High income country studies only(n = 38) (c). Upper middle-income countries only (n = 8) (d). Lower middle-income countries only (n = 4) (e). Studies with corresponding Subnational GBD estimates only (n = 18) (f) Studies which are reported as informing the GBD 2021 model only (n = 45). Blue solid line = 95% limits of agreement, Red dashed line = difference population-based stroke register crude incidence—GBD crude incidence, PBSR = population-based stroke register, ASIR = age-standardized incidence rate.

Bland–Altman plots of matched crude incidence rates are displayed in Figure 1.

Mean population-based stroke register crude incidence was 193.8/100,00 (SD: 75.8) and mean GBD crude incidence was 181.9/100,00 (SD: 79.6). Overall, 11 (22.0%) of the GBD estimates, accounting for UIs, matched population-based stroke register crude incidence rates. Twenty-seven (54.0%) of GBD estimates were lower than primary data rates and 12 (24.0%) of GBD estimates were higher than primary data rates. GBD crude estimates were lower overall (−11.9), in HICs (−17.5s) and in LMICs (−43.6) and higher in UMICs (30.2). Concordance and correlation were 0.67 and 0.68 overall, 0.66 and 0.68 for HICs, 0.59 and 0.77 for UMICs, and 0.03 and 0.97 for LICs respectively. GBD crude incidence estimates were lower in Africa (−24.9), Europe (−12.5), Latin America and Caribbean (−19.0), North America (−24.7), and Oceania (−41.2) and higher in Asia (15.8) compared to population-based crude incidence measurements. Concordance and correlation were 0.50 and 0.69 in Asia, 0.63 and 0.66 in Europe, 0.26 and 0.55 in Latin America and Caribbean, and 0.08 and 0.34 in Oceania. Concordance and correlation were not calculated for Africa and North America due to low sample size.

The 95% limits of agreement, the range where differences in the two measurement methods for crude stroke incidence are expected to fall were from −134.1/100,00 to 110.2/100,000 overall.

## Age-standardized stroke incidence

**Figure 2.**
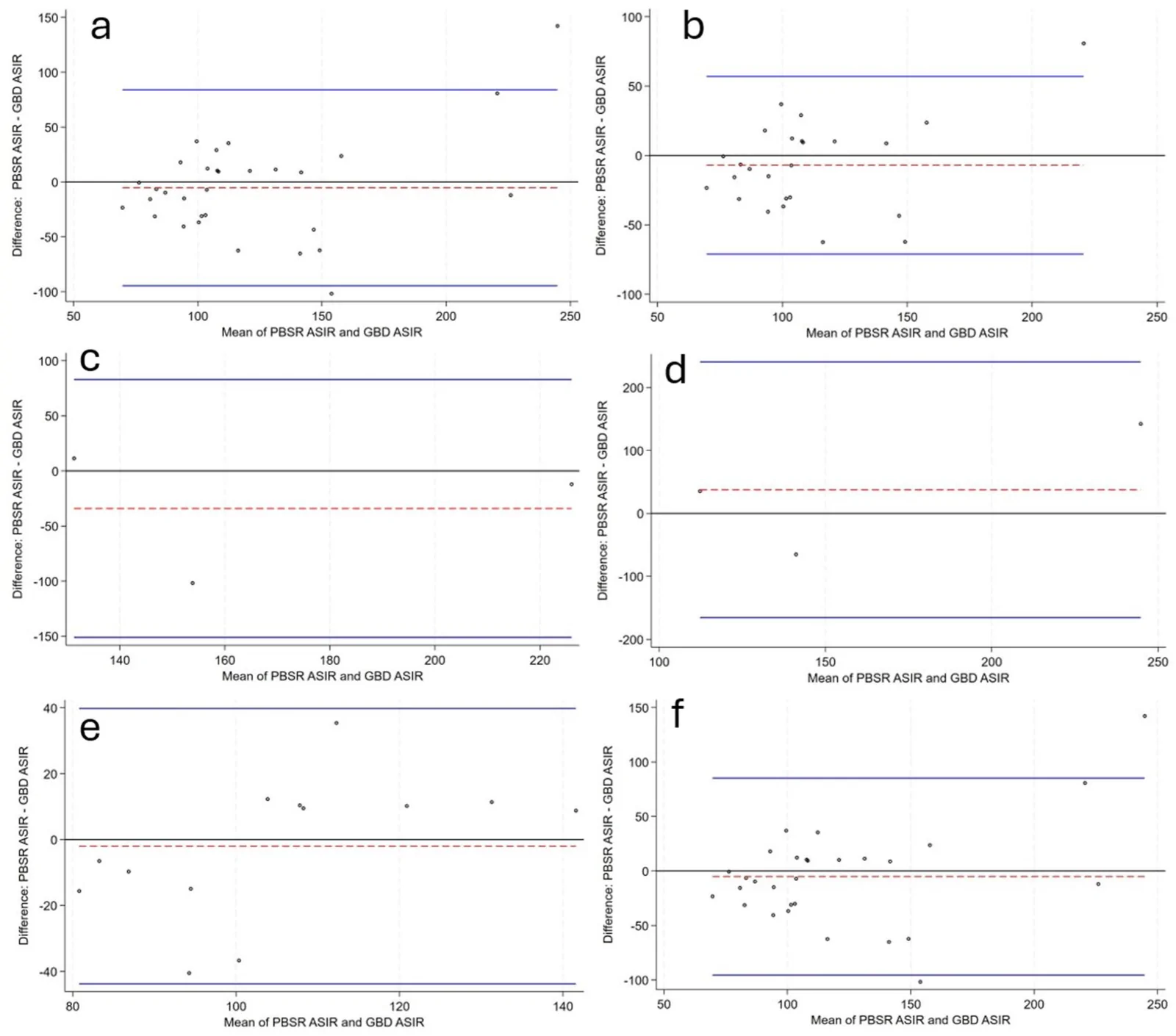
Bland–Altman plots of crude incidence from population-based stroke registers compared to GBD crude incidence estimates per 100,000 population and 95% limits of agreement: (a) all studies (n = 50); (b) high-income country studies only (n = 38); (c) Upper middle-income countries only (n = 8); (d). Lower middle-income countries only (n = 4); (e) studies with corresponding subnational GBD estimates only (n = 18); and (f) studies which are reported as informing the GBD 2021 model only (n = 45). Blue solid line = 95% limits of agreement, Red dashed line = mean of the difference population-based stroke register crude incidence—GBD crude incidence, PBSR = population-based stroke register, ASIR = age-standardized stroke incidence.

Age-standardized incidence rates, standardized to the WHO World Standard population, were available for 31 of the population-based stroke register incidence rates and are compared to 31 age-standardized rates from the GBD 2021 model, matched by year and location (see Table 2). Mean population-based stroke register age-standardized incidence was 118.6/100,000 (SD: 56.3) and mean GBD age-standardized incidence was 124.0 (SD: 40.4). Overall, 9 (29.0%) of GBD estimates age-standardized, accounting for UIs, matched population-based estimates. Concordance and correlation for age-standardized incidence rates were 0.56 and 0.59 overall, 0.59 and 0.63 for HICs, 0.12 and 0.17 for UMICs, and 0.25 and 0.42 for LMICs. The 95% limits of agreement, the range where differences in the two measurement methods for age-standardized stroke incidence are expected to fall were from −94.6 to 84.1/100,000. Bland–Altman plots of matched age-standardized incidence rates are displayed in Figure 2.

**Table 2.** Concordance, correlation, and 95% limits of agreement for age-standardized stroke incidence rates by country income group, region, studies with corresponding subnational GBD estimates only and studies reported as informing GBD estimates only.

	Number of PBSR: count (%)	PBSR age-standardized stroke incidence (SD) per 100,000 population	GBD age-standardized incidence (SD) per 100,000 population	Difference in agreement	PBSR age-standardized incidence falls within GBD UI: count (%)	Lin’s concordance coefficient	Pearson’s correlation coefficient	95% limits of agreement per 100,000 population
All	31	118.6 (56.3)	124.0 (40.4)	6.5	9 (29.0%)	0.56	0.59	-94.6 to 84.1
High-income countries	25	106.5 (41.5)	113.5 (30.4)	7.0	7 (28%)	0.59	0.63	-71.0 to 57.0
UMICs	3	153.3 (60.2)	187.4 (55.3)	46.1	2 (66.7%)	0.12	0.17	-151.1 to 82.9
LMICS	3	184.8 (114.0)	147.3 (45.7)	-37.5	0 (0.0%)	0.25	0.42	-165.7 to 240.7
Africa	2	212.3 (146.6)	173.7	-38.6	0 (0.0%)	-	-	-
Asia	3	126.3 (21.7)	145.5 (75.1)	19.2	1 (33.3%)	-0.34	-0.79	-161.7 to 123.3
Europe	17	116.6 (54.4)	125.6 (43.4)	9.0	5 (29.4%)	0.71	0.74	-80.6 to 62.5
Latin America and Caribbean	4	111.8 (22.4)	104.9 (19.6)	-6.9	1 (25.0%)	0.21	0.22	-44.6 to 58.4
North America	2	100.5 (17.7)	110.4 (11.1)	9.9	1 (50.0%)	-	-	-
Oceania	3	81 (17.1)	94 (15.7)	13.0	1 (33.3%)	0.35	0.52	-44.7 to 18.7
Studies with corresponding Subnational GBD estimates only	13	104.1 (25.6)	106.1 (15.3)	2.0	7 (53.9%)	0.49	0.56	-43.8 to 39.8
Studies reported as informing GBD estimates	29	118,3 (58.3)	123.5 (40.5)	6.5	9 (31.0%)	0.54	0.57	-95.7 to 85.3

PBSR = population-based stroke register.

## GBD uncertainty intervals

**Figure 3.**
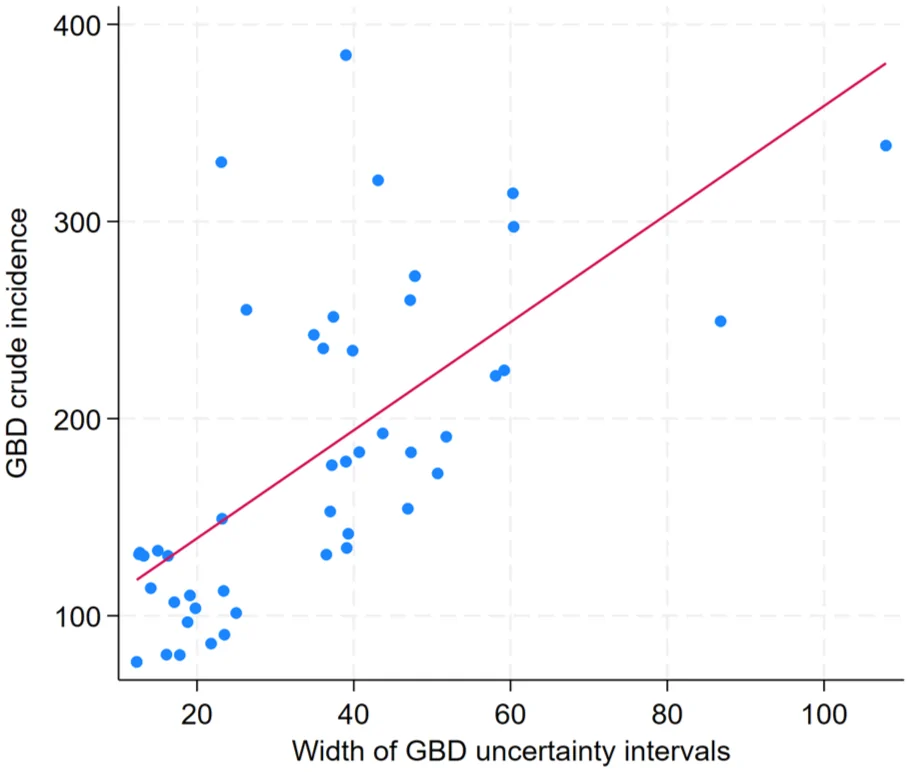
Scatterplot of value of GBD crude incidence and width of GBD crude incidence uncertainty intervals. Solid red line = Line of best fit.

The number of primary data sources to inform GBD first-ever ischemic stroke incidence was 73, ranging from 27 (42.9%) sources from Western Europe, to 0 sources from Oceania, Southeast Asia, Caribbean, Andean Latin America, Central Latin America, Central Sub-Saharan Africa, and Western Sub-Saharan Africa (see Table 3). Mean width of UIs was 27.8 (SD: 20.0), ranging from 82.4 in East Asia with 5 data sources to 10.6 in Western Sub-Saharan Africa with 0 data sources. The mean width of 95% UI was significantly lower 14.7 (SD: 2.4) in regions with no data sources than in countries with one or more data sources 34.3 (SD: 5.7), unpaired t-test p = 0.03. For the 50 GBD crude incidence estimates in our analysis, width of GBD UIs was significantly associated with the value of the crude incidence estimate, paired t-test, p < 0.0001, see Figure 3.

**Table 3.** Width of GBD uncertainty intervals vs number of data sources used to inform GBD model by region for first-ever ischemic stroke.

Region	Ischemic stroke incidence sources	GBD first-ever ischemic stroke incidence	GBD upper UI	GBD lower UI	Width of UI
Western Europe	27	126.6	141	113.3	27.7
North Africa and Middle East	9	76.6	86.9	67.5	19.4
Australasia	8	99.7	113.2	87.3	25.9
East Asia	5	210.7	255.8	173.4	82.4
Eastern Europe	5	205.6	243.4	171.7	71.7
High-income Asia pacific	4	176	200.9	152.3	48.6
South Asia	4	47.9	56.2	40.7	15.5
Central Europe	3	192.2	218.3	167	51.3
Southern Latin America	2	79.8	91.2	69.5	21.7
Tropical Latin America	2	81	96	68.1	27.9
Central Asia	1	100.6	114.6	88.4	26.2
High-income North America	1	93.5	110.8	79	31.8
Eastern Sub-Saharan Africa	1	37.4	42.8	32.1	10.7
Southern Sub-Saharan Africa	1	59.8	70	50.6	19.4
Oceania	0	39.4	44.9	34.1	10.8
Southeast Asia	0	96	110.5	83.3	27.2
Caribbean	0	75.3	85.2	66.2	19
Andean Latin America	0	40.5	46.4	35.3	11.1
Central Latin America	0	48.9	56.1	42.3	13.8
Central Sub-Saharan Africa	0	34.6	40.3	29.6	10.7
Western Sub-Saharan Africa	0	39.1	44.5	33.9	10.6
Total	73				Mean: 27.8 (SD: 20.0)

## Discussion

This is the first study to report calibration of the GBD 2021 stroke incidence model. Concordance and correlation for crude stroke incidence were 0.67 and 0.68 overall. The wide 95% limits of agreement, −110.2/100,00 to 134.1/100,000, demonstrate the discrepancies in agreement between the two methods. Overall, 11 (22.0%) GBD estimates, accounting for UIs, matched population-based stroke register crude incidence rates. Given that GBD is a principal source of global stroke estimates, the overall discrepancies in agreement, should cause reflection in the use and interpretation of these estimates. Concordance decreased by country income group, 0.66 for HICs, 0.59 for UMICs, and 0.03 for LMICs, highlighting that more caution should be applied in utilizing these estimates in LMICs.

The analysis of age-standardized incidence reported similar findings. Concordance and correlation were 0.56 and 0.59, respectively. Overall, 9 (29.0%) of GBD age-standardized incidence estimates, accounting for UIs, matched population-based stroke register rates. The 95% limits of agreement were −94.6/100,000 to 84.1/100,000. Concordance for age-standardized incidence rates was highest in HICs, 0.59, and lower in UMICs 0.12 and LMICs 0.25. The potential causes of the discrepances in agreement are discussed in the following paragraph.

This study compares the concordance and correlation of two difference sources of stroke incidence estimates. Population-based stroke registers provide stroke incidence measurements of high accuracy for specific locations, which may not be representative of the national population. Conversely, the GBD model, is a single model which estimates total health burden globally for 288 causes in 204 countries and territories and 811 subnational locations, of which stroke is just one cause. GBD 2021 incorporates data from population-based stroke registers, including 90% of the population-based stroke registers in our sample. The differences in incidence rates could be explained by a number of reasons. First, population-based stroke register studies are most frequently critiqued for under ascertainment of cases,^17,18^ which would lead to population-based stroke register study rates being lower than GBD rates, however our results show that GBD crude incidence estimates were generally lower than crude incidence from population-based stroke registers. Second, population-based registers may over ascertain cases, due to definition and diagnosis of stroke, availability of neuroimaging and through cause of death ascertainment. To mitigate this in our study, we included only population-based stroke registers that used the WHO definition of stroke, prospective design, and where stroke was diagnosed by a stroke physician or neurologist and excluded registers which relied on case ascertainment through medical record coding or International Classification of Disease coding. Third, the catchment population of population-based stroke registers may not be representative of the national population, subgroup analysis including only studies where more specific subnational geographical GBD estimates were available marginally improved crude incidence (n = 18) concordance (0.67 vs 0.71) but not age-standardized incidence (n = 13) concordance (0.53 vs 0.49). In this subgroup, only one third of population-based stroke register incidence rates fell with the GBD UIs. Fourth, the population-based register data may not be used to inform the GBD 2021 model, however subgroup analysis limited to population-based stroke registers included as GBD 2021 sources, did not significantly improve correlation or concordance. Fifth, the differences could be due to additional data sources and modeling used by GBD 2021. GBD strives to use all available data (465 data sources are reported as informing the 2021 GBD model), including hospital-based data, which are then incorporated into the Bayesian meta-regression tool DisMod-MR. GBD sources include routine health service stroke data and hospital-based stroke studies, which are then adjusted to account for under-ascertainment and underreporting. Under ascertainment of hospital-based stroke registers in low resource settings have demonstrated much higher rates of under ascertainment compared to high resource settings.^19^ Finally, the differences in rates could be due to the overarching GBD modeling process, which prioritizes internal consistency. GBD calculates an “all cause envelope,” the total health burden globally, divided into hierarchies with level one causes categorized as communicable diseases, non-communicable diseases, and injuries, then further levels providing more disease and subtype specific causes, all contributing to the total all-cause health burden. These hierarchies are mutually exclusive, no overlap between causes and collectively exhaustive capture all causes. As the sum of cause-specific mortality and cause-specific impairment, must equal all-cause mortality and all-cause impairment, then we speculate that differences in stroke estimates from GBD compared to population-based stroke registers could arise from over or under-estimation of the health burden of other non-communicable diseases. For example, if the health burden of ischemic heart disease is over estimated, then estimates of stroke could be constrained in order to fit the all-cause envelope.

A key principle of the GDB 2021 study is that “it is important to convey to users the strength of the evidence for each quantity through the reporting of uncertainty intervals”^20^ and “larger uncertainty intervals can result from limited data availability, small studies, and conflicting data, while smaller uncertainty intervals can result from extensive data availability, large studies, and data that are consistent across sources.”^21^ In this analysis, the width of GBD UIs was associated with the absolute number of strokes reported by the GBD model, low crude incidence rates were associated with narrow UIs and high crude incidence rates with wide UIs, paired t-test (p < 0.001). It appears, in our study that width of GBD UIs are primarily driven by the absolute number of strokes reported by the GBD model, rather than the number and quality of sources used to inform the model. Indeed, accounting for the number of GBD sources used by region, reported counterintuitive findings, that the width of GBD UIs for first-ever ischemic stroke was significantly narrower in regions with no data sources contributing to the GBD estimates than in regions contributing one or more data sources (p = 0.03). As an example, the GBD 2021 crude stroke incidence UIs are twice as wide for the United Kingdom, where several population-based incidence measurements are available, 127.66–156.69 (width: 29.3), compared to in 81.04–95.00 (width: 14.0) in Sierra Leone, where no population-based measurements of crude stroke incidence are available. Similar questions have been raised for GBD breast and cervical cancer incidence UIs^11^ and for mortality rates due to diabetes.^12^ Further clarity and explanation of the calculation of GBD UIs should be provided in GBD manuscripts and GBD should consider clearly displaying the availability and quality of source data per estimate.

## Limitations

This analysis focuses only on stroke incidence; it did not attempt to assess GBD estimates of stroke deaths and DALYs. The study is limited in its ability to explore reasons behind the discrepancies found due to the complex nature of the GBD methodology and the lack of GBD model calibration results provided. Another study evaluating reliability of GBD 2019 estimates for prevalence of musculoskeletal conditions applied GRADE^22^ to evaluate data inputs used in GBD 2019, and found certainty of estimates to be low, mainly due to risk of bias and indirectness.^23^ In this study, a limited assessment of study quality was made using standardized criteria,^8,15^ with no application of the GRADE system. While we applied more stringent inclusion criteria for population-based stroke registers, we did include population-based registers with lower than the ideal 90% neuroimaging rate, this potentially could have led to over ascertainment of stroke due to the inclusion of stroke mimics within the numerator. Population-based stroke register measurements may not be representative of the national population, leading to discrepancies of agreement; however, our subgroup analysis where GBD subnational data was available did not significantly improve concordance. This study reports whether population-based stroke register incidence rates fell with GBD UIs; however, we highlight that GBD width appears to be related to absolute number of strokes predicted, providing narrower UIs for locations with lower crude stroke incidence, the use of different methods of calculating UIs by GBD would impact our findings. This analysis could not assess concordance and correlation for the many countries where population-based stroke register data is not available and where GBD estimates potentially have the most utility, new high-quality population-based stroke registers in these locations will be vital to measure stroke incidence and assess accuracy of GBD estimates.

## Conclusion

Accurate calculation of stroke incidence is critical to understanding burden of disease and monitoring efficacy and progress of primary prevention strategies. Our findings demonstrate limited concordance and correlation in crude and age-standardized stroke incidence rates between population-based stroke registers and the GBD 2021 model, with lower accuracy for non HICs. As GBD is increasingly used to measure global progress on stroke,^24^ reporting of the GBD model calibration for stroke incidence estimates alongside interpretable and intuitive reporting of uncertainty is essential. High-quality population-based stroke register studies, including in LICs and LMICs, should remain a priority.^25^

## Supplemental Material

sj-docx-1-wso-10.1177_17474930261423425 – Supplemental material for The concordance and correlation of Global Burden of Disease stroke incidence rates with stroke incidence rates from population-based stroke registers Supplemental material, sj-docx-1-wso-10.1177_17474930261423425 for The concordance and correlation of Global Burden of Disease stroke incidence rates with stroke incidence rates from population-based stroke registers by Daniel Youkee and Charles DA Wolfe in International Journal of Stroke
