# Supplementary material for: The concordance and correlation of Global Burden of Disease stroke incidence rates with stroke incidence rates from population-based stroke registers
Source: Int J Stroke. 2026 Feb 3;21(8):1140–9. doi: 10.1177/17474930261423425 (PMC13601702; doi:10.1177/17474930261423425)
Supplement: Supplementary file 1 [file sj-docx-1-wso-10.1177_17474930261423425.docx]

**Supplementary Table one**

| PBSR Country | Year | PBSR crude incidence | GBD crude incidence | GBD Upper UI | GBD Lower UI | PBSR Age-standardized incidence (WHO World Standard Population) | GBD age-standardized incidence | GBD upper UI | GBD lower UI | PBSR included as GBD source | Neuroimaging rate for studies <90% neuroimaging |
| --- | --- | --- | --- | --- | --- | --- | --- | --- | --- | --- | --- |
| Australia (Adelaide) | 2009-2010 | 161 | 114 | 121 | 107 | 76 | 76.6 | 81.4 | 72.0 | Yes |  |
| Australia (Melbourne) | 1996-1997 | 206 | 133 | 140 | 125 | 100 | 107.1 | 112.9 | 101.2 | Yes |  |
| Australia (Perth) | 2000-2001 | 128 | 130 | 138 | 122 | 67 | 98.3 | 104.0 | 91.9 | Yes |  |
| Belarus (Grodno) | 2001-2003 | 222 | 314 | 344 | 284 | 220 | 232 | 253.1 | 211.9 | Yes | 30.3% |
| Brazil (Matão)* | 2003-2004 | 108 | 101 | 115 | 90 | 137 | 125.6 | 141.8 | 111.3 | Yes |  |
| Chile (Iquique) | 2000-2002 | 74 | 107 | 116 | 98 | 86 | 117 | 126.9 | 108.2 | Yes |  |
| Chile (Ñuble) | 2015-2016 | 180 | 110 | 120 | 101 | 122 | 92.9 | 100.9 | 84.7 | No |  |
| China (Beijing) | 2000 | 178 | 178 | 199 | 160 | - | - | - | - | Yes |  |
| China (Changsha) | 2000 | 201 | 178 | 199 | 160 | - | - | - | - | Yes |  |
| China (Shanghai) | 2000 | 160 | 178 | 199 | 160 | - | - | - | - | Yes |  |
| Croatia (Varaždin county) | 2007-2009 | 276 | 255 | 268 | 241 | 169.6 | 145.9 | 152.6 | 138.9 | No |  |
| Estonia (Tartu) | 2001-2003 | 223 | 252 | 270 | 233 | 125 | 168.4 | 179.5 | 157.4 | No | 19-90% |
| Finland (Espoo-Kauniainen) | 1989-1991 | 220 | 235 | 255 | 215 | - | - | - | - | Yes |  |
| Finland (Turku) | 1992 | 451 | 243 | 260 | 225 | - | - | - | - | Yes |  |
| France (Dijon) | 2000-2006 | 114 | 131 | 137 | 125 | 58 | 81.3 | 85.0 | 77.6 | Yes |  |
| French West Indies (Martinique) | 2004-2008 | 147 | 132 | 138 | 126 | - | - | - | - | Yes |  |
| French West Indies (Martinique) | 1998-1999 | 167 | 130 | 137 | 124 | 102 | 84.0 | 88.0 | 79.9 | Yes |  |
| Georgia (Tbilisi) | 2000-2003 | 165 | 321 | 342 | 298 | 103 | 240.7 | 254.4 | 226.2 | Yes |  |
| Germany (Erlangen) | 1994-1996 | 175 | 236 | 254 | 218 | 85 | 147.4 | 158.6 | 136.7 | Yes |  |
| Greece (Arcadia) | 1993-1995 | 344 | 260 | 285 | 238 | - | - | - |  | Yes |  |
| India (Ludhiana) * | 2012-2013 | 140 | 80 | 89 | 73 | 130 | 94.6 | 104.5 | 85.1 | Yes | 35.4% |
| India (Mumbai) * | 2005-2006 | 145 | 80 | 90 | 72 | - | - | - | - | Yes | 89.2% |
| Iran (Mashhad) * | 2006-2007 | 139 | 86 | 98 | 76 | - | - | - | - | Yes |  |
| Ireland (North Dublin) | 2005-2006 | 165 | 97 | 107 | 88 | 118 | 81.0 | 89.1 | 73.8 | Yes |  |
| Italy (Belluno)* | 1992-1993 | 224 | 154 | 180 | 133 | 110 | 97.7 | 112.0 | 85.3 | Yes |  |
| Italy (L’Aquila)* | 1994 | 275 | 183 | 208 | 160 | 113 | 103.5 | 115.7 | 92.3 | Yes |  |
| Italy (Sesto Fiorentino) * | 2004-2006 | 170 | 193 | 216 | 172 | - | - | - | - | No |  |
| Italy (Valley d' Aosta)* | 2004-2005 | 223 | 183 | 205 | 164 | 82 | 91.7 | 101.6 | 83.2 | Yes |  |
| Italy (Valley d' Aosta) * | 2004-2008 | 212 | 176 | 196 | 159 | 80 | 86.5 | 95.2 | 78.7 | Yes |  |
| Italy (Vibo Valentia) * | 1996 | 179 | 153 | 172 | 135 | 87 | 101.9 | 113.5 | 90.6 | Yes |  |
| Japan (Iwate State) * | 2004-2008 | 290 | 297 | 329 | 268 | 146 | 137.2 | 150.3 | 125.2 | Yes |  |
| Lithuania (Kaunas) | 2004 | 223 | 384 | 405 | 366 | - | - | - | - | No |  |
| New Zealand (Auckland) | 2011-2012 | 147 | 104 | 114 | 94 | - | - | - | - | Yes |  |
| New Zealand (Auckland) | 2002-2003 | 158 | 113 | 125 | 101 | - | - | - | - | Yes |  |
| Norway (Innherred) | 1994-1996 | 312 | 249 | 295 | 209 | - | - | - | - | No |  |
| Poland (Warsaw) | 2005 | 106 | 172 | 198 | 147 | - | - | - | - | Yes |  |
| Portugal (Porto, rural) | 1998-2000 | 305 | 272 | 296 | 248 | 261 | 180.2 | 193.8 | 165.9 | Yes |  |
| Portugal (Porto, urban) | 1998-2000 | 268 | 272 | 296 | 248 | 118 | 180.2 | 193.8 | 165.9 | Yes |  |
| Sweden (Lund-Orup)* | 2001-2002 | 194 | 222 | 252 | 194 | - | - | - | - | Yes |  |
| Sweden (Örebro)* | 1999-2000 | 314 | 225 | 255 | 196 | 126 | 115.8 | 130.9 | 102.2 | Yes |  |
| Tanzania (Dar-es-Salaam, urban) | 2003-2006 | 108 | 77 | 83 | 70 | 315.9 | 173.7 | 187.0 | 158.8 | Yes | 79.1% |
| Tanzania (Hai, rural) | 2003-2006 | 95 | 77 | 83 | 70 | 108.6 | 173.7 | 187.0 | 158.8 | Yes | 79.1% |
| Ukraine (Uzhhorod) | 1999-2000 | 281 | 339 | 398 | 290 | - | - | - | - | Yes |  |
| United Kingdom (East Lancashire)* | 1994-1995 | 158 | 191 | 218 | 166 | 74 | 114.5 | 128.8 | 101.0 | Yes |  |
| United Kingdom (London)* | 2004-2006 | 70 | 90 | 102 | 79 | - | - | - | - | Yes |  |
| United Kingdom (Oxfordshire)* | 2002-2004 | 145 | 142 | 162 | 123 | 73 | 88,6 | 100.8 | 77.4 | Yes |  |
| United Kingdom (Scottish Borders) | 1998-2000 | 280 | 330 | 342 | 318 | - | - | - | - | Yes |  |
| United Kingdom (South London)* | 1995-1996 | 130 | 131 | 150 | 114 | 82 | 118.7 | 134.7 | 104.0 | Yes |  |
| USA (Barbados) | 2001-2002 | 140 | 149 | 161 | 138 | 88 | 118.1 | 127.4 | 109.2 | Yes |  |
| USA (Greater Cincinnati)* | 1999 | 193 | 134 | 156 | 116 | 113 | 102.6 | 118.5 | 89.4 | Yes |  |

*Table of population-based stroke registers, location and year of study, PBSR crude incidence, GBD crude incidence and uncertainty intervals, PBSR Age-standardized incidence (WHO World Standard Population), GBD age-standardized stroke incidence and uncertainty intervals and whether the PBSR was included as a source for GBD 2021. *Indicates subnational GBD estimates were available and used. PBSR=Population based stroke register*
